# Spontaneously hypertensive rats display reduced microglial activation in response to ischemic stroke and lipopolysaccharide

**DOI:** 10.1186/1742-2094-9-114

**Published:** 2012-05-30

**Authors:** Deborah De Geyter, Wendy Stoop, Tine Zgavc, Sophie Sarre, Yvette Michotte, Jacques De Keyser, Ron Kooijman

**Affiliations:** 1 Department of Pharmacology, Center for Neuroscience, Vrije Universiteit Brussel, Laarbeeklaan 103, Brussels, Belgium; 2 Department of Pharmaceutical Chemistry and Drug Analysis, Center for Neuroscience, Vrije Universiteit Brussel, Laarbeeklaan 103, Brussels, Belgium; 3 Department of Neurology, Universitair Ziekenhuis Brussel, Center for Neuroscience, Vrije Universiteit Brussel, Laarbeeklaan 103, Brussels, Belgium; 4 Department of Neurology, University Medical Center Groningen, Groningen, The Netherlands

**Keywords:** Focal cerebral ischemia, Endothelin-1, Hypertension, Glial cells, Blood flow

## Abstract

**Background:**

For successful translation to clinical stroke studies, the Stroke Therapy Academic Industry Round Table criteria have been proposed. Two important criteria are testing of therapeutic interventions in conscious animals and the presence of a co-morbidity factor. We chose to work with hypertensive rats since hypertension is an important modifiable risk factor for stroke and influences the clinical outcome. We aimed to compare the susceptibility to ischemia in hypertensive rats with those in normotensive controls in a rat model for induction of ischemic stroke in conscious animals.

**Methods:**

The vasoconstrictor endothelin-1 was stereotactically applied in the vicinity of the middle cerebral artery of control Wistar Kyoto rats (WKYRs) and Spontaneously Hypertensive rats (SHRs) to induce a transient decrease in striatal blood flow, which was measured by the Laser Doppler technique. Infarct size was assessed histologically by Cresyl Violet staining. Sensory-motor functions were measured at several time points using the Neurological Deficit Score. Activation of microglia and astrocytes in the striatum and cortex was investigated by immunohistochemistry using antibodies against CD68/Iba-1 and glial fibrillary acidic protein.

**Results and conclusions:**

The SHRs showed significantly larger infarct volumes and more pronounced sensory-motor deficits, compared to the WKYRs at 24 h after the insult. However, both differences disappeared between 24 and 72 h. In SHRs, microglia were less susceptible to activation by lipopolysaccharide and there was a reduced microglial activation after induction of ischemic stroke. These quantitative and qualitative differences may be relevant for studying the efficacy of new treatments for stroke in accordance to the Stroke Therapy Academic Industry Round Table criteria.

## Background

Many clinical trials with neuroprotective drugs in patients with acute ischemic stroke have yielded disappointing results [1,2]. This failure may be due to the use of inadequate animal models. To facilitate translation to the clinic, the Stroke Therapy Academic Industry Round table (STAIR) has developed criteria for more clinically relevant research in animal models of ischemic stroke. The current paper evaluates a model for translational research fulfilling the STAIR criteria. An important requirement is testing of drugs in animal models with a co-morbidity, such as diabetes or hypertension. We have chosen hypertension, because it is a modifiable risk factor for both ischemic and hemorrhagic stroke [3]. In addition, more than 50% of stroke patients show an acute hypertensive response, which appears to be related to a poor clinical outcome [4,5], and pre-existing hypertension can exacerbate this response to acute stroke [6]. Two different rat strains with spontaneous hypertension have often been used as an animal model for ischemic stroke. Spontaneously hypertensive rats (SHRs) were developed in the Kyoto School of Medicine in Japan from an outbred Wistar Kyoto male rat (WKYR) with marked elevation of blood pressure mated to a female with slightly elevated blood pressure. The other model [7] is the stroke-prone spontaneously hypertensive rat (SPSHR), which develops spontaneous strokes. This model exhibits increased sensitivity to experimental stroke which may, in addition to genetically determined hypertension, also be due to additional genetic factors [8]. Although additional genetic factors exacerbating the effects of experimental stroke cannot be excluded in SHRs, we preferred to use this strain to study the effects of hypertension on experimental ischemic stroke, because increased susceptibility to a reduction in blood flow has been shown to be related to the development of hypertension in this strain. An increased infarct size in SHRs compared to normotensive controls was observed in early and late stage hypertensive rats, but not in pre-hypertensive rats [9].

Induction of ischemic stroke in conscious animals is another crucial requirement for preclinical testing according to the STAIR criteria, because most anesthetics are neuroprotective [10-12]. We have chosen to use the endothelin (Et)-1 model [13] for induction of a local reduction in blood flow in the middle cerebral artery (MCA), because this model allows induction of vasoconstriction followed by reperfusion in conscious rats.

In this study, we addressed the differences in susceptibility to the induction of brain ischemia in conscious SHRs and normotensive controls using the Et-1 model. Since glial cells, such as microglia and astrocytes, respond to ischemia and damage of neural tissues, we especially focused on differences in activation of these cells during stroke.

## Material and methods

### The et-1 rat model

Protocols for animal experiments were designed according to the European Guidelines on Animal Experimentation and approved by the Ethical Committee for Animal Experimentation of the Vrije Universiteit Brussel (VUB). Male albino WKYRs and SHRs (Charles River Laboratories, Saint Germain sur l’Arbresle, France) were housed in groups of four and allowed to acclimatize to their new environment for one week until they weighed 275 to 300 g.

**Figure 1:**
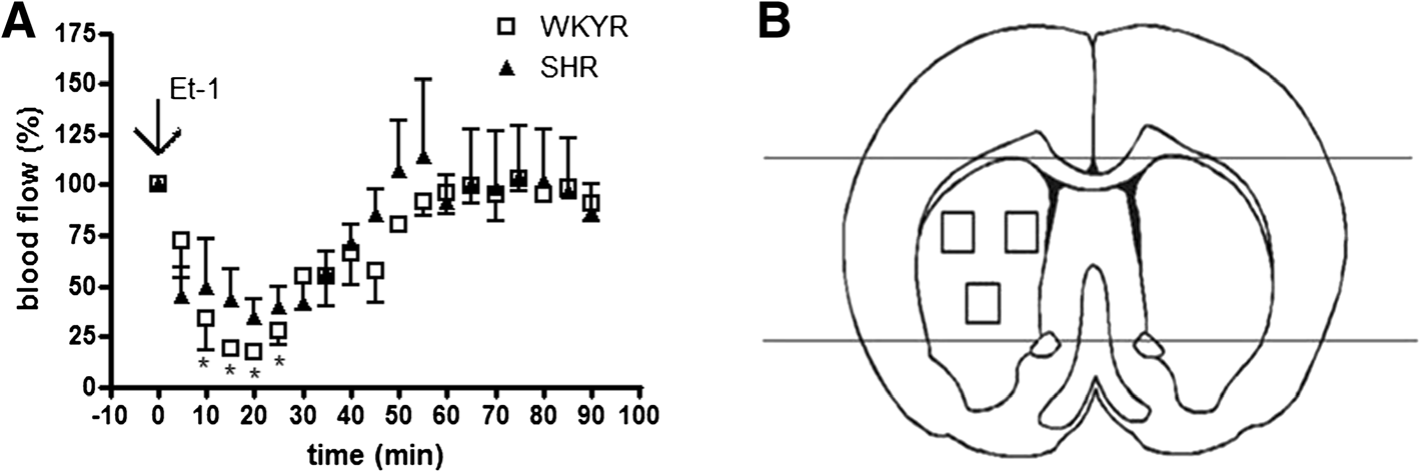
**A: Differences in blood flow reduction in the striatum of anesthetized WKYRs and SHRs.** Shown are differences in blood flow reduction in the striatum of anesthetized WKYRs and SHRs after administration of 400 pmol Et-1. There were no differences between the two strains. *Significantly different from baseline levels. Differences between SHR and WKY were not significant for all time points as assessed by the unpaired Student’s *t*-test. (n = 4 for every group). **B**: Fixed fields in the striatum and cortex used for assessment of CD68 and GFAP expression. The three fixed fields are indicated in the striatum. Each has an area of 1 mm^2^. Cells are counted in the three separate fields and the results are expressed as mean number of cells/mm^2^. For the cortex, cells are counted between the reference lines. To determine the level of GFAP expression, the relative intensity is measured in the whole striatum and in the cortical region between the reference lines.

Surgery was performed as described previously by Van Hemelrijck *et al.*[14]. Briefly, 24 h before Et-1 administration, animals were anesthetized by an intraperitoneal injection of a mixture of ketamine (75 mg/kg) and diazepam (3.5 mg/kg) and placed on a stereotactic frame in order to position a guide in the vicinity of the MCA. The stereotactic coordinates were determined according to the atlas of Paxinos and Watson [15] (coordinates relative to bregma: AP +0.9 mm, L +5.0 mm and V +2.8 mm). After surgery, rats received an intraperitoneal injection of the analgesic ketoprofen (5 mg/kg) and were allowed to recover overnight having free access to tap water and standard laboratory chow. The next day, the guide was replaced by a microdialysis probe of which the membrane was removed (CMA, 3 mm, Solna, Sweden) through which Et-1 (Sigma, St. Louis, MO, USA), dissolved in an iso-osmotic Ringers’ solution, was perfused into the freely moving animals at a rate of 1 μl/minute during six minutes. Reduction in striatal blood flow by Et-1 is shown in Figure 1A. Sham operated rats were injected with the same volume of Ringer’s solution.

### Neurological deficit score

In order to measure motoric and sensory deficits, the Neurological Deficit Score (NDS) was measured as described in Garcia *et al.*[16,17]. In this test, six parameters, including symmetry in the movement of the forelimbs, spontaneous activity, climbing, body proprioception, response to vibrissae touch and fore paw outstretching, were evaluated, resulting in a total score ranging from 3 to 18. The score is inversely related to the neurological deficit. These parameters were assessed 24 h before injection of Et-1 and at 1, 6, 24, 48 and 72 h after the insult.

### Histology and immunohistochemistry

Rats were sacrificed at 24 or 72 h after the insult by intraperitoneal injection of an overdose of sodium pentobarbital and then transcardially perfused for 5 minutes with 0.9% sodium chloride followed by perfusion for 5 minutes with a 4% phosphate-buffered paraformaldehyde solution (pH 7.42) in order to fixate the brains. Next, animals were decapitated and brains were post-fixed in paraformaldehyde. Brain slices of 50 μm were made and kept at 4 °C in a preservative buffer solution (0.01 M phosphate buffered saline (PBS) with 0.01% sodium azide).

To determine the infarct size, a series of equidistant 50 μm sections, comprising every fourth section, from 4.70 to 1.80 mm from bregma was collected, mounted onto gelatin coated slices and stained with 0.5% Cresyl Violet acetate. The infarct areas were outlined on digitized images, after which the surface of these areas was calculated using Image J software (NIH, version 1.43). Subsequently, the infarct volume (v) in mm³ was estimated from the surface areas (a) according to the Cavalieri principle for estimation of volumes [18] using the following formula: v = d × Σa, where d is the distance between the upper (rostral) surfaces of two consecutive analyzed sections.

Activation of glial cells was assessed by immunohistochemistry on 50 μm thick brain slices mounted on 3-aminopropyltriethoxylane (APES)-coated slides using antibodies against glial fibrillary acidic protein (GFAP) as a marker for astrocytes and antibodies against ED-1 (CD68, a lysosomal glycoprotein) and Iba-1 (ionized calcium binding adaptor molecule 1) for assessing microglial activation. CD68 selectively stains activated microglia and macrophages, whereas Iba-1 stains activated microglia more intensely than surveiling microglia. Due to its expression on the cell surface Iba-1 also allows morphological identification of activated microglia. Inducible nitric oxide synthase (iNOS) was used as a marker of oxidative stress and inflammation. The density of CD68- or Iba-1-stained cells in the striatum and cortex was calculated in three coronal sections from each rat, located between 0.90 mm and 0.20 mm anterior to bregma to avoid interference caused by implantation of the probe and expressed as the mean number of cells per mm^2^. On CD68 stained sections, the density of CD68 positive cells were assessed and on Iba-1 stained sections, the densities of both round-shaped cells and ramified microglia with thickened processes were measured. In striatal sections, three different areas were defined (Figure 1B). In each area the number of cells was counted by using a counting grid and evaluated by WCIF Image J (NIH, version 1.37). In the cortex, the total number of cells was assessed on the surface between the lines crossing the center of the anterior commissures and the top of the striatum (Figure 1B). The surface of this area was calculated by Image J software (NIH, version 1.43). GFAP and iNOS staining were quantified as followed: (intensity value contralateral striatum (or cortex) – intensity value ipsilateral striatum (or cortex)) using Image J software (NIH, version 1.43) in the whole striatum and in the cortical region between the reference lines. Calculating the difference between the ipsi-and contralateral side is necessary to correct for interassay variability in GFAP staining [17] (Figure 1B).

#### CD68, iba-1 and iNOS-staining

After preincubation at room temperature with 3% H_2_0_2_, 0.1% Triton X-100 and pre-immunized goat serum (1:5 dilution, Sigma), brain slices were incubated overnight at 4 °C with polyclonal mouse anti-CD68 (1:500 in normal goat serum/PBS 1/5, AdB Serotec, Dusseldorf, Germany, catalog number: MCA341R), anti-Iba-1 (1:1,000 in normal goat serum/PBS 1/5,Wako Pure Chemicals Industries, Osaka, Japan, catalog number: 019–19741) or anti-iNOS (1:3,500 in normal goat serum/PBS 1/5, Millipore, Temecula, CA, USA, catalog number: AB5382). The next day, the slices were incubated for 4 h at room temperature with a sheep anti-mouse IgG for the ED-1 staining (1:100 in normal goat serum/PBS 1/5, GE Healthcare UK Limited, Little Chalfont, Buckinghamshire, UK, catalog number: NA931V) or donkey anti-rabbit IgG for Iba-1 and iNOS staining (1:100 in normal goat serum/PBS 1/5, GE Healthcare UK Limited, Little Chalfont, Buckinghamshire, UK, catalog number: NA934V). Antibody binding was visualized using the diaminobenzidine substrate chromogen kit (Dako Cytomation, Glostrup, Denmark). Between all incubation steps, washing steps were performed for five minutes using PBS/0.1% Tween-20. For the iNOS staining, slices were counterstained with 0.5% Cresyl Violet acetate after assessing iNOS expression levels.

#### GFAP staining

After preincubation at room temperature with 0.1% H_2_0_2,_ normal donkey serum (1:10 dilution) and 0.1% Triton X-100, brain slices were incubated for 24 h with polyclonal rabbit anti-GFAP (1/10,000 in donkey serum/0.5% PBS Dako, Glostrup, Denmark, catalog number: Z0334). Next, slices were incubated for 1 h with a simple stain MAX peroxidase-labeled anti-rabbit IgG (histofen, Nichirei Biosciences Inc, Tokyo, Japan, catalog number: 414181 F). Antibody binding was visualized using the diaminobenzidine substrate chromogen kit (Dako, Glostrup, Denmark). Between all incubation steps, washing steps were performed for five minutes using PBS. Brain slices where mounted on APES coated slices.

### Laser Doppler flowmetry in anesthetized animals

For these experiments, it was not possible to work with conscious animals. Since the sevoflurane used is neuroprotective [11,12], a higher dose of Et-1 was necessary to produce the same infarct as in awake animals. We considered 400 pmol as the optimal dose because of the infarct size and NDSs were comparable to the 200 pmol without anesthesia (data not shown). After implantation of the cannula for Et-1 administration (see section 1.1.), a second probe was implanted in the striatum (coordinates relative to bregma AP +1.2 mm, L +2.4 mm and V +5 mm). After 24 h, rats were anesthetized with 4% sevoflurane and oxygen 0.8 L/min into a transparent chamber, placed on a heating blanket and maintained under anesthesia using 2.5 to 3% sevoflurane delivered with oxygen 0.8 L/minutes via a facemask during the entire experiment. The Et-1 injection was injected as described in section 2.1 and the probe in the striatum was replaced by the Laser (Laser Flow Blood perfusion monitor probe, TSI, Inc., Shoreview, MN, USA). Ten minutes after reaching stable baseline levels, 400 pmol Et-1 was administered and the blood flow was measured in the striatum for 90 minutes. Cerebral blood flow values are expressed as a percentage of the mean pre-stroke baseline level.

### The lipopolysaccharide rat model for neuroinflammation

Surgery for probe positioning in the striatum (coordinates relative to bregma AP +1.2 mm, L +2.4 mm and V +2 mm) was performed as described for Et-1 injection. One day after surgery the guide was replaced by a probe as described in section 1.1 and 2, 10 or 40 μg lipopolysaccharide (LPS) (*E. coli*, Sigma, MO, USA, catalog number: L7895) dissolved in 0.9% saline was injected into the striatum at a rate of 0.5 μl/minute during four minutes. Sham operated rats were injected with the same volume of saline. Twenty four hours after LPS administration, rats were sacrificed and 50 μm brain slices were made.

### Statistics

All data are expressed as mean ± SEM and statistical significance between two groups was tested using the unpaired Student’s *t*-test. Significant differences between sham and Et-1 treated rats (WKYRs and SHRs) were assessed using a one-way ANOVA followed by the Bonferroni *post-hoc* test. In the LPS experiments, a one-way ANOVA following by the Dunnett's Multiple Comparison *post-hoc* test was used. Differences in NDS were assessed using the Mann–Whitney *U*-Test. Repeated measures one-way ANOVA followed by the Dunnett's post-test was applied to detect significant differences between pre- and post-stroke levels of striatal blood flow. Statistical analysis was done using Graphpad Prism (version 4.03, GraphPad Software, San Diego, CA, USA). All tests were performed at the 0.05 level of significance.

## Results

### Induction of ischemic stroke in conscious WKYRs and SHRs

**Figure 2:**
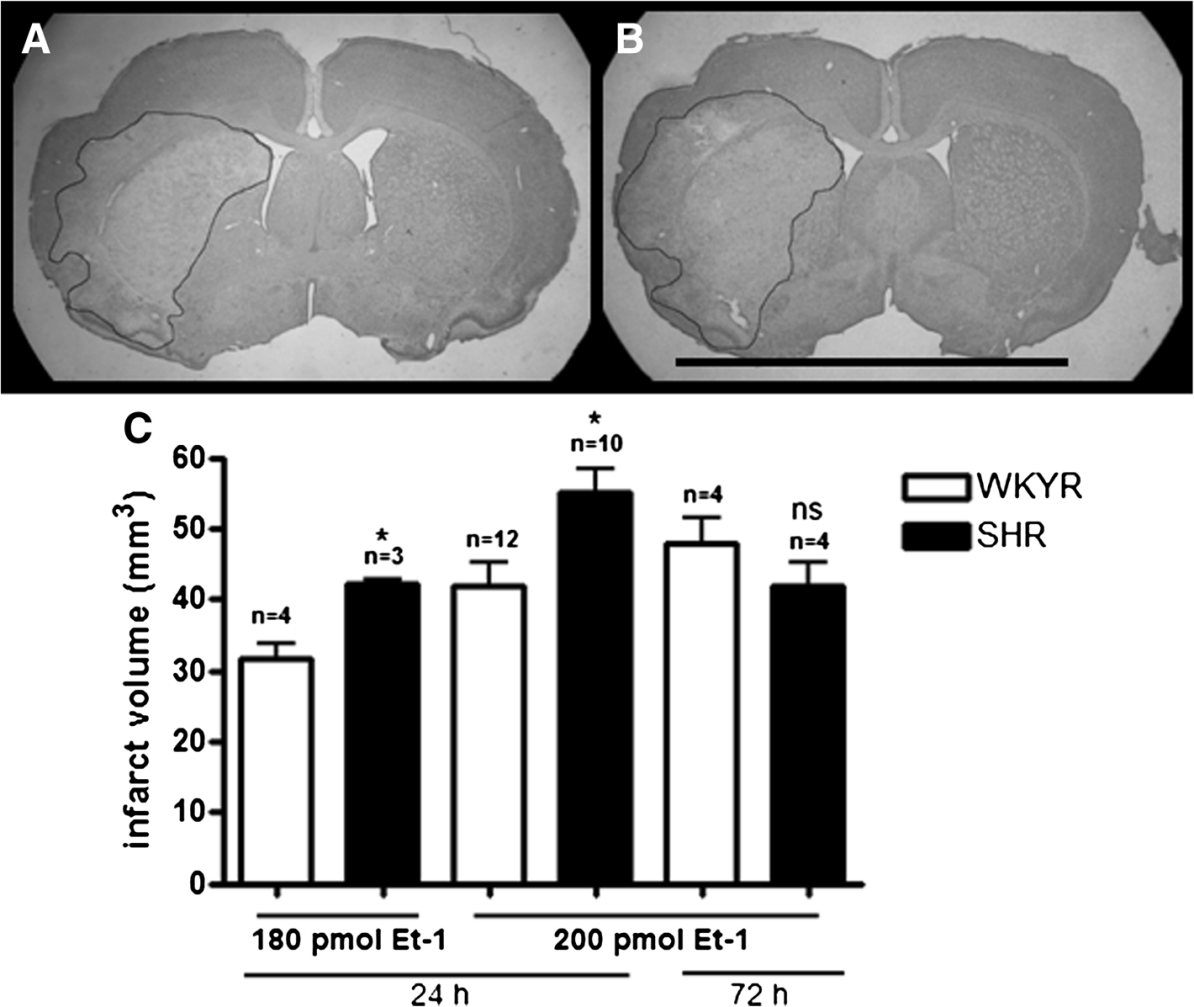
**Differences in infarct volume between WKYRs and SHRs.** Representative micrographs (scale bar 1 cm) of infarcts in a WKYR and SHR are shown in **A** and **B**, respectively. Brain slices were stained with Cresyl Violet 24 h after induction of the insult with 200 pmol Et-1. Graph C shows the differences in infarct volume between WKYRs and SHRs assessed after induction of focal cerebral ischemia with 180 or 200 pmol Et-1. *Significant difference between WKYRs and SHRs. ns: no significant difference between WKYRs and SHRs.

**Figure 3:**
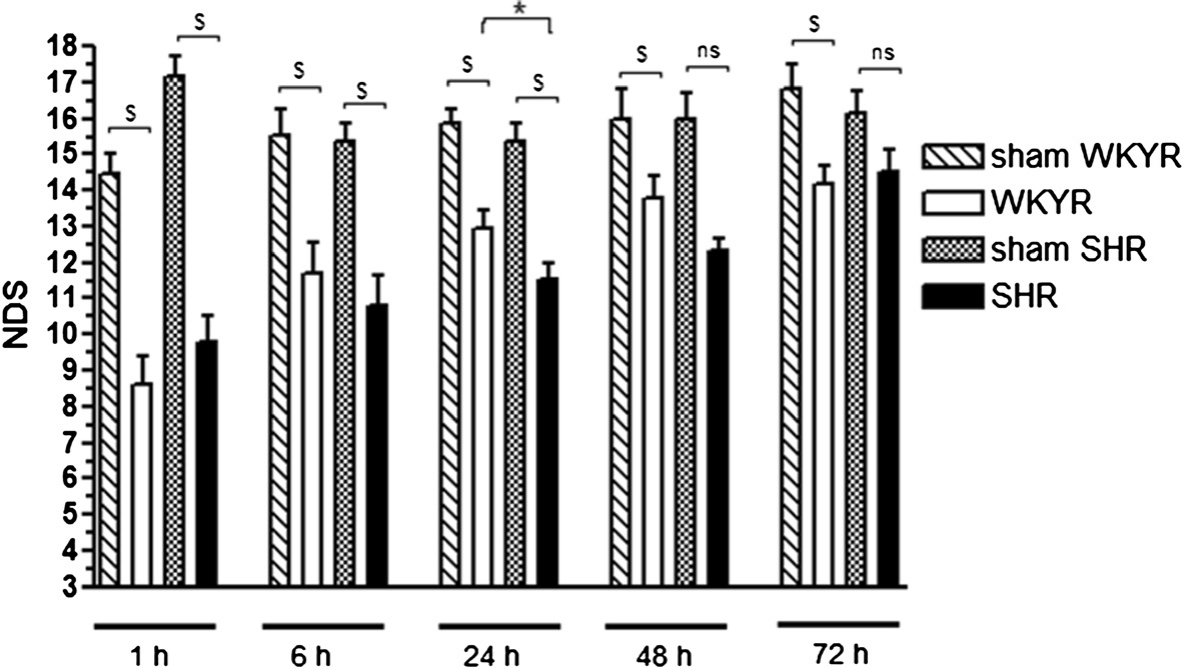
**Neurological Deficit Scores in sham operated rats and Et-1 treated WKYRs and SHRs.** Rats were injected with 200 pmol Et-1 and the NDS was assessed at different time points. At 24 h, there was a significant difference between the NDS of the two strains.. *Significant difference between WKYRs and SHRs. ^$^ Significant difference between sham and Et-1 treated animals. ns: no significant difference between WKYRs and SHRs. Group sizes were n = 6 to 13 at 1 h, n = 5 to 10 at 6 h, n = 13 to 15 at 24 h, n = 3 to 5 at 48 h and n = 4 to 6 at 72 h.

We first performed a dose-range finding study in order to induce an infarct situated in the striatum and cortex of conscious WKYRs. Bogaert *et al.*[13] found that a dose of 120 pmol Et-1 was necessary to produce such an infarct in normal Wistar rats. However, in WKYRs, 120 pmol did not induce an infarct or significant sensory-motor deficits (data not shown). Figure 2C shows that a dose of 180 pmol Et-1 induced an infarct of 31.7 ± 1.9 mm³. However, the infarct was restricted to the striatum. In contrast, a dose of 200 pmol induced an infarct of 41.8 ± 3.3 mm³ (Figure 2C), which was present in both the striatum and cortex (Figure 2A). At these doses there was no mortality, whereas injection of 240 pmol Et-1 in WKYRs resulted in a mortality of 57% (data not shown). A dose of 200 pmol also produced a significant neurological deficit for at least three days in the WKYRs (Figure 3) and was, therefore, selected for further studies.

Figure 2C also reveals that the SHRs initially produced significantly larger infarcts in response to injection of 180 and 200 pmol Et-1 compared to the WKYRs. However, the difference between these strains was not observed at 72 h after the insult, because the SHRs showed a tendency towards a decrease in infarct size after 24 h. In both strains, Et-1 injection evoked a significant reduction in NDS compared to sham operated animals at 1, 6, 24 h after the insult, which means 200 pmol induced sensory-motor deficits until at least one day after stroke. The higher infarct volume in the SHRs at 24 h after the insult coincided with a significantly lower NDS compared to normotensive controls (Figure 3). However, at later time points the reduction in NDS became smaller and was not significant. This phenomenon is in line with the decrease in infarct size after 72 h.

### Et-1-induced reduction in striatal blood flow in WKYRs and SHRs

To determine whether the increased infarct size in SHRs was due to a stronger reduction in blood flow, we measured the blood flow in the striatum of both strains. Every five minutes, the blood flow was assessed and the reduction in blood flow was expressed as a percentage of baseline levels. In the WKYRs, Et-1 induced a significant reduction in blood flow compared to baseline at 10, 15, 20 and 25 minutes after the insult. Blood flow was already decreased after 5 minutes and remained around 20% of normal levels between 15 and 25 minutes after the insult. After 60 minutes, blood flow levels were back to normal. In the SHRs, blood flow also decreased within 5 minutes after administration of Et-1, but did not drop under 30% of the baseline levels. Normal blood flow levels in SHRs were reestablished after 50 minutes. Although WKYRs showed a stronger decrease in blood flow in response to Et-1, the differences between the two strains did not reach significance at any time point (Figure 1A). Taken together, these findings indicate that SHRs are more sensitive to reduction of striatal blood flow resulting in an increased infarct size.

### Microglia in SHRs are less susceptible to activation in both the et-1 model for ischemic stroke and the LPS model for neuroinflammation

Microglia are considered as the resident macrophages of the brain and are the first cells in the brain responding to ischemic injury gaining phagocytotic properties. They are essential for neuroprotection, but can also induce detrimental processes, such as neuroinflammation and excessive infiltration of leukocytes [19-22]. In order to compare the activation of microglia in normotensive and hypertensive rats, we performed immunohistochemistry using anti-CD68 as a marker for phagocytotic microglia and macrophages.

**Figure 4:**
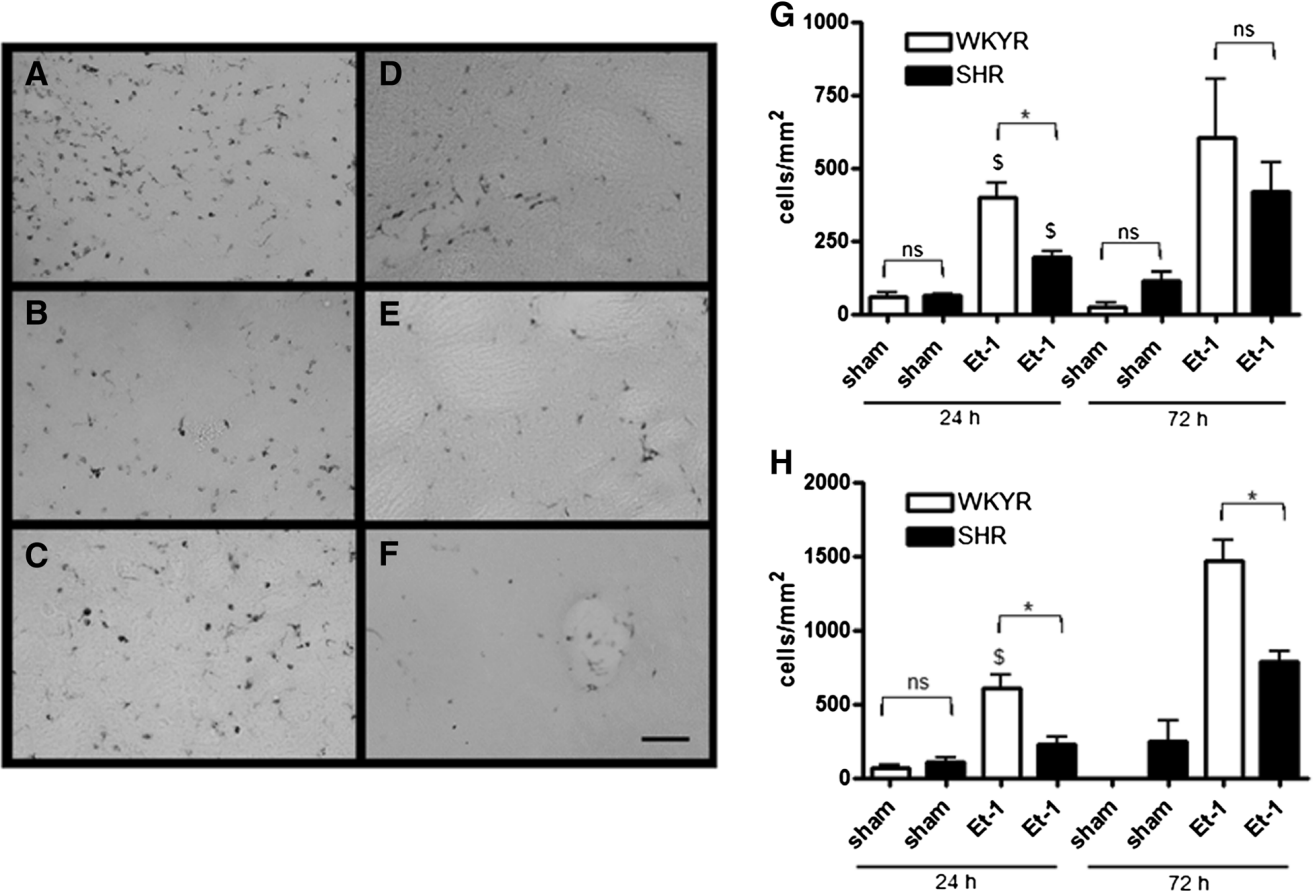
**Levels of CD68 positive cells in WKYRs and SHRs after Et-1 injection.** Brain slices from WKYRs (n = 4) and SHRs (n = 4) treated with 200 pmol Et-1 were stained with anti-CD68 and the density of CD68^+^ cells was assessed in three fixed regions of the striatum and one region in the cortex (see Figure 1B) . Representative micrographs from two fixed regions in the striatum (**A** and **D**, upper left region; **B** and **E** upper right region) and one region in the cortex (**C** and **F**, in the center of the indicated region) are shown for WKYRs (A-C) and SHRs (D-F). Scale bar = 50 μm. Densities of CD68^+^ cells in the striatum (G) and cortex (H) are provided for both strains. The expression of CD68 remained low in the sham operated groups (n = 3 to 4 for the WKYR’s and n = 4 for the SHRs). *Significant difference between WKYRs and SHRs. ^$^ Significant difference between sham and Et-1 treated animals. ns: no significant difference between WKYRs and SHRs.

CD68 positive cells were only found within the infarct zone. The density of these cells was low around the boundaries and stronger at the center of the infarct. The highest levels were found in the transition zone between striatum and cortex. This can be observed in the micrograph of Figure 4A taken from the upper left area of the striatum indicated in Figure 1B. It appears that most cells are present on the left side of the micrograph, which is closest to the cortex. CD68 expression in sham operated animals was relatively low in the ipsilateral hemisphere (Figure 4G, H) and absent in the contralateral hemisphere of all groups (data not shown). Although the SHRs exhibited higher infarct volumes at 24 h, they expressed 50% less CD68 positive cells in the striatum at this time point (Figure 4G, D, E). At 72 h, the density of CD68 positive cells in the striatum was also reduced in the SHRs, although this difference did not reach statistical significance. Similar results were observed in the cortex where the expression of CD68 was significantly lower in the SHRs (Figure 4F) compared to WKYRs (Figure 4C) at both 24 and 72 h (Figure 4H).

**Figure 5:**
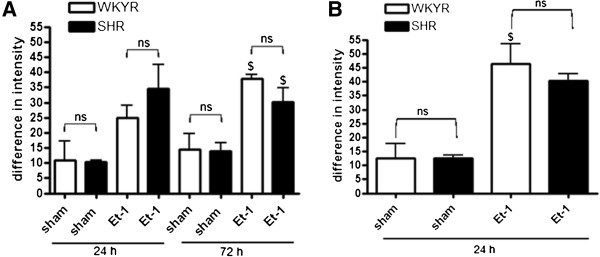
**GFAP expression levels in WKYRs and SHRs after Et-1 injection**. Comparison of GFAP expression levels in the striatum (**A**) and cortex (**B**) of WKYRs and SHRs after induction of focal cerebral ischemia with 200 pmol Et-1. There is no difference between the two rats at every time point (n = 3 to 4 for the sham WKYRs and n = 4 to 5 for the Et-1 treated WKYRs and n = 3 to 4 for the sham SHRs and n = 4 to 5 for the Et-1 treated SHRs). *Significant difference between WKYRs and SHRs. ^$^ Significant difference between sham and Et-1 treated animals. ns: no significant difference between WKYRs and SHRs.

Next, we addressed the activation of astrocytes using an antibody against the commonly used activation marker GFAP. At 24 and 72 h after Et-1 administration, there was no significant difference between GFAP expression levels in the striatum and cortex (no data at 72 h) of normotensive and hypertensive rats. (Figure 5A, B).

**Figure 6:**
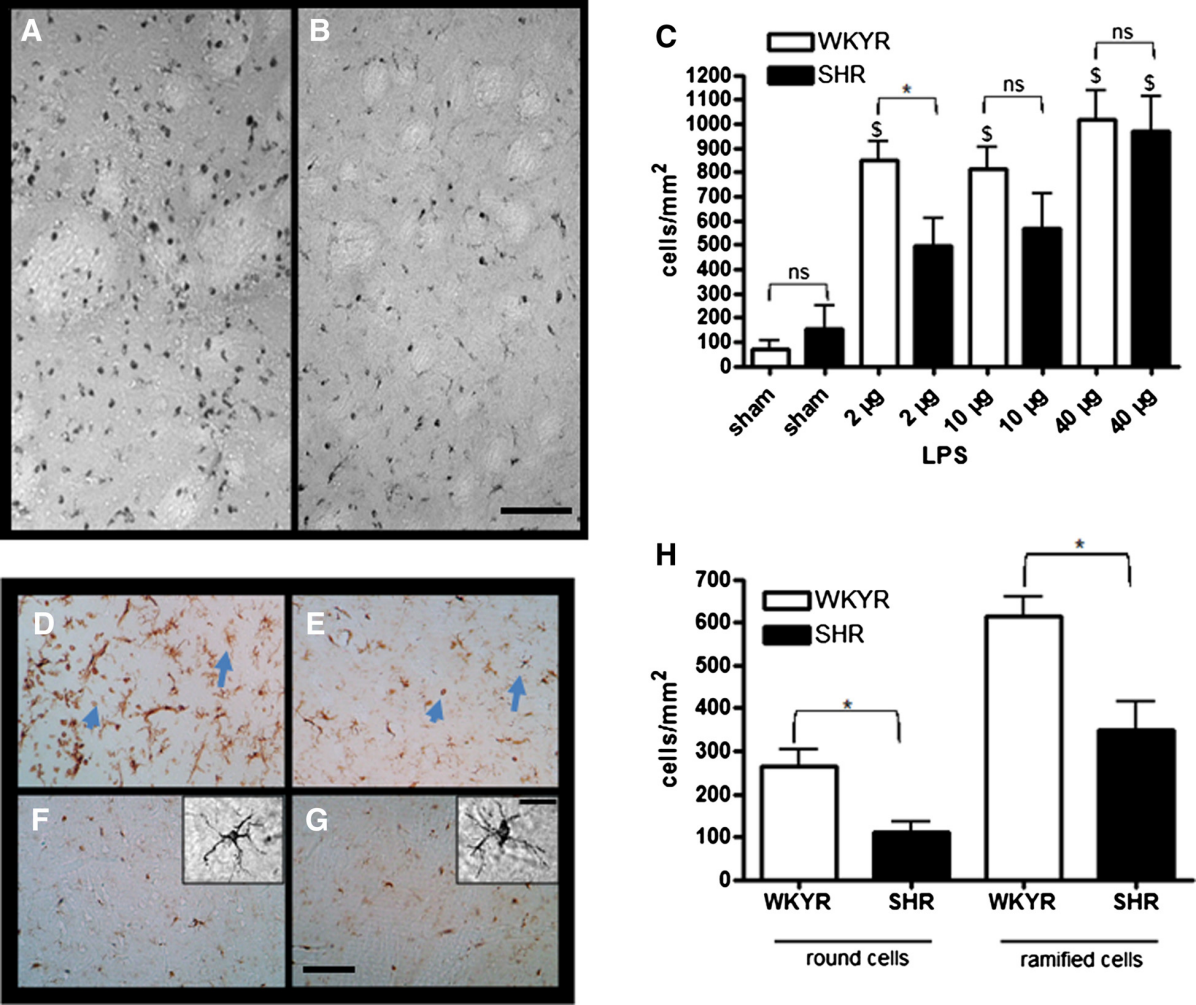
**CD68 (A-C) and Iba-1 expression levels (D-H) in WKYRs and SHRs 24 hrs after injection of LPS.** The expression of CD68 is shown in a micrograph taken from a representative region in the striatum from WKYRs (**A**) and SHRs (**B**) and quantitative results are presented in the graph **C**. (The number of animals per group were n = 3 for the sham WKYRs and n = 4 for the sham SHRs; n = 4 for the LPS-treated groups). The expression of Iba-1 is shown for the ipsilateral (**D, E**) and contralateral hemispheres (**F, G**) for WKYRs (D, F) and SHRs (E, G). Graph H depicts quantitative differences in Iba-1 expression in the striatum of WKYRs and SHRs at 24 h after injection of 2 μg LPS (n = 4 for the all groups). *Significant difference between WKYRs and SHRs. ^$^ Significant difference between sham and Et-1 treated animals. ns, no significant difference between WKYRs and SHRs. Scale bar A, B = 50 μm; scale bar D-G = 50 μm, scale bar insert = 20 μm.

**Figure 7:**
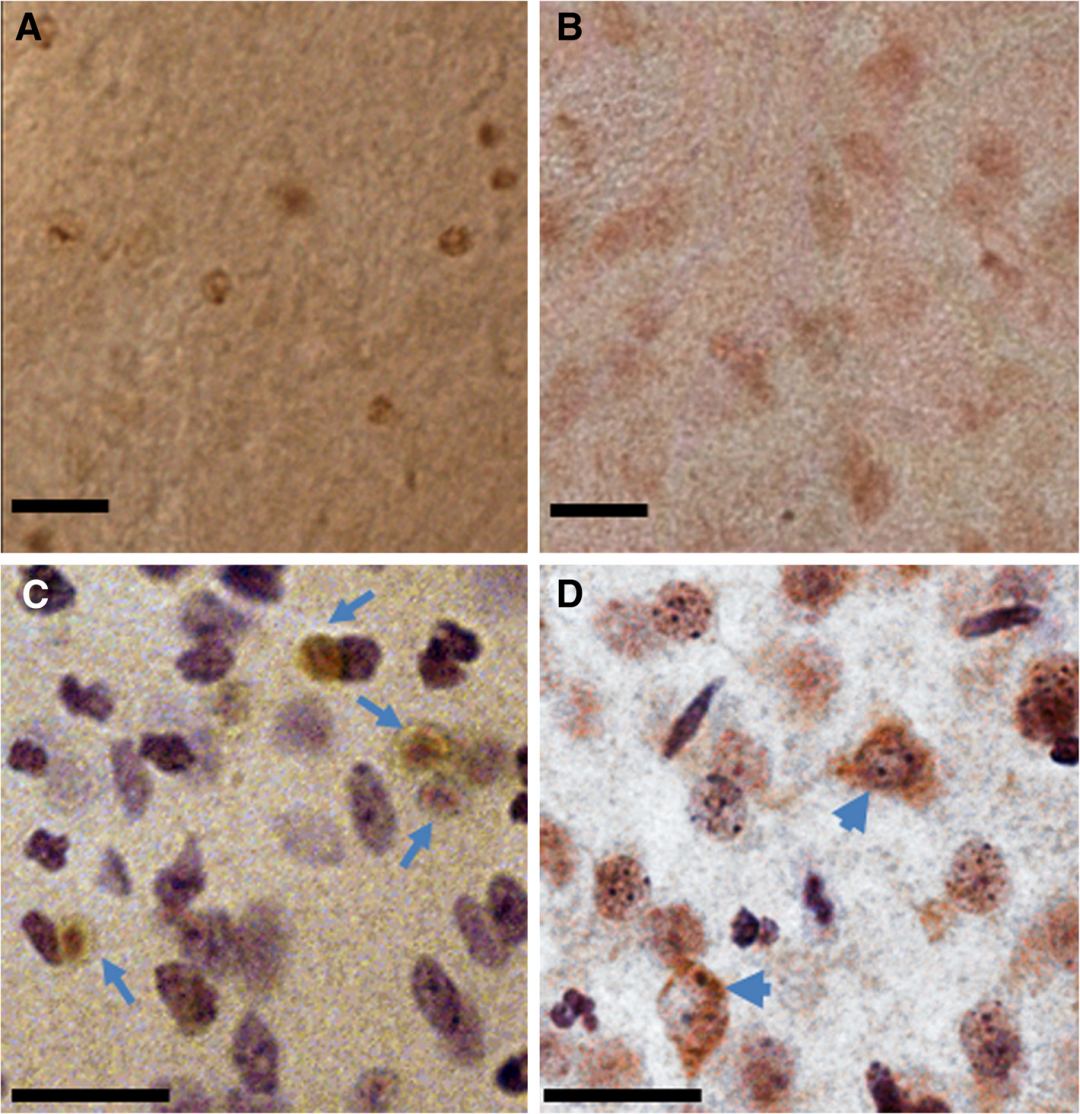
**iNOS expression levels in WKYRs and SHRs 24 hrs after injection of 2 μg LPS.** Brains slices from WKYRs (**A**) and SHRs (**B**) were stained with an anti-iNOS antibody. **C** (WKY) and **D** (SHR) represent similar micrographs counterstained with Cresyl Violet and taken at a larger magnification. Cells expressing iNOS are indicated by arrows (C) or arrow heads (neurons in 7D). Scale bar = 20 μm.

Our observation that SHRs exhibit higher infarct volumes in combination with a reduced activation of microglia, suggests that microglia in SHRs are less susceptible to activation than their counterparts in normotensive rats. To test this hypothesis, we assessed the effects of LPS on microglial activation in both strains. LPS is a bacterial endotoxin acting via direct stimulation of the Toll-like receptor (TLR), which is commonly expressed in microglia and responsible of their activation by several endogenous ligands including intracellular components of dead cells including heat-shock proteins and oligonucleotides [23,24]. By assessing microglial responsiveness using LPS as a stimulus, indirect effects via differences in blood flow reduction are completely excluded. As shown in Figure 6C, it appears that microglia of both strains equally responded to injection of a high dose of LPS (40 μg). However, when 2 μg of LPS was injected, the density of CD68 positive cells was markedly lower in SHRs compared to WKYRs (Figure 6C). Figure 6A and B show a lower density of CD68 positive cells in the SHRs (Figure 6B) compared to the WKYRs (Figure 6A) in the striatum after injection of 2 μg LPS. To confirm the idea that the reduced density of CD68 positive cells was due to impaired activation of microglia, another marker for activated microglia was tested. Antibodies against Iba-1 stain activated microglia more intensely than their non-activated counterparts and allow morphological identification of activated microglia. After labeling the slices of the rats that were injected with 2 μg of LPS, three types of microglia were distinguished: ramified microglia with thin processes (Figure 6F, G), ramified microglia with thickened processes and shortened endpoints (arrows in Figure 6D, E) and round-shaped phagocytic cells (arrow heads in Figure 6D, E). The first subset, representing resting microglia, was mainly observed at the contralateral side of WKYRs (Figure 6F) and SHRs (Figure 6G). At the ipsilateral side, we measured the density of activated microglia with thick processes and the round-shaped cells. We found that the SHRs showed significantly reduced numbers of both subtypes in the ipsilateral side (Figure 6H). To further address possible consequences on oxidative stress and neuroinflammation, we evaluated iNOS expression 24 h after injection of 2 μg LPS using an antibody against iNOS. The difference in total staining intensity between the two rat strains was not significant although it seemed that the SHRs (14.92 ± 5.68) display lower levels of iNOS expression than WKYRs (22.12 ± 4.32) (data not shown, n = 4 for both groups). However, the most remarkable difference between the two strains was that LPS injection in SHRs led to iNOS expression in cells that were larger than the iNOS-expressing cells in the normotensive controls (Figure 7A, B). Indeed, counter staining with Cresyl Violet revealed a remarkable difference in the predominant cell type expressing iNOS. In WKYRs cells expressing iNOS were not stained with Cresyl Violet (arrows in Figure 7C), while in SHRs iNOS expressing cells were identified as neurons (arrow heads in Figure 7D).

**Figure 8:**
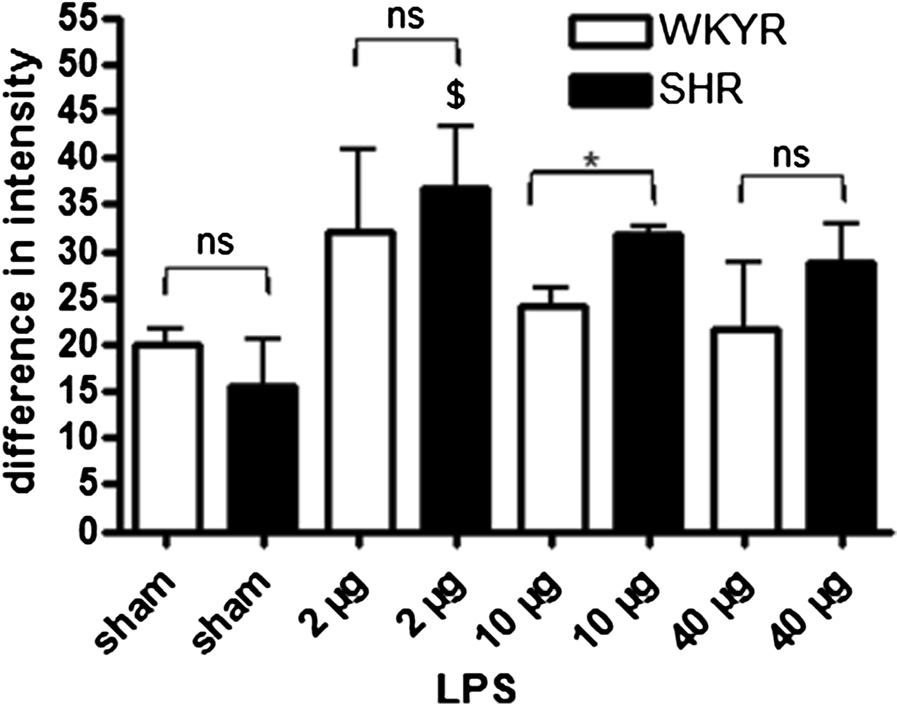
**GFAP expression levels in WKYRs and SHRs 24 h after injection of LPS.** Comparison of GFAP expression levels in the striatum of WKYRs and SHRs after injection of 2, 4 and 40 μg LPS in the striatum (n = 3 for the sham WKY rats and n = 4 to 5 for the Et-1-treated WKY rats and n = 4 for the sham SHRs and n = 4 for the Et-1 treated SHRs). * Significant difference between WKY and SHR. ^$^ Significant difference between sham and Et-1 treated animals. ns: no significant difference between WKY rats and SHRs.

In contrast to the observations for microglia we found that SHRs displayed a stronger activation of astrocytes in response to LPS as assessed by anti-GFAP staining (Figure 8).

## Discussion

Using different animal models for ischemic stroke, several groups showed that hypertensive rats are more vulnerable to cerebral ischemia than normotensive rats. Hom *et al.*[9] found a 40% increase in infarct volume in 10- and 15-week old SHRs compared with age-matched WKY controls in a 4 h filament model. Barone *et al.*[25] observed that SHRs have higher cortical infarctions compared to the controls and Marks *et al.*[26] found a significant increase in infarct volume in SPSHRs compared to the WKYRs. These experiments were all performed in MCA occlusion models in which the blood flow in the MCA was mechanically blocked by intrusion of an intraluminal suture (filament model) [27,28].

In order to meet an important part of the STAIR criteria for preclinical research on stroke, we used the Et-1 model to reduce cerebral blood flow in conscious animals. In this study, we addressed the differences in responses to cerebral ischemia between conscious hypertensive SHRs and normotensive WKYRs. We found that SHRs exhibit larger infarct volumes compared to their controls 24 h after administration of 180 and 200 pmol Et-1. Sensory-motor behavior was tested with the NDS [16,17]. We tested the animals at 1, 6, 24, 48 and 72 h after the insult. Sham operated rats showed a small decrease in NDS, probably due to surgery and implantation of the guide. In both rat strains we observed a significant decrease in NDS compared to the sham operated rats until 24 h after the insult. The NDS of hypertensive rats, displaying increased infarct sizes, was significantly lower than the NDS of WKY controls at 24 h. Since 200 pmol induces larger infarct sizes, while having a relatively small effect on striatal blood flow, we postulate that the hypertensive model allows induction of larger infarct sizes and maybe stronger deficits by increasing the dose of Et-1. Using the normotensive model, stronger effects can not be reached due to the high mortality rate at higher doses.

It should be noted that the increased infarct size and the reduction in NDS in SHRs compared to WKYRs disappeared between Day 1 and Day 3 after the insult. A possible explanation might be that the reduction in blood flow in SHRs leads to a bigger infarct with a relatively larger penumbral area at 24 h after ischemia, which can be (partially) recovered.

The most commonly used rodent model for focal cerebral ischemia is blocking the blood flow in MCA by insertion of an intraluminal suture [27,28]. In this model reperfusion takes place directly after removal of the suture. In contrast, in the Et-1 rat model, reduction in blood flow in the MCA is induced by injection of the vasoconstrictor Et-1 and reperfusion occurs spontaneously as a result of elimination of Et-1. In this model, strain differences in sensitivity to Et-1 or in degradation rate of Et-1 could lead to differences in the reduction of the blood flow and, subsequently, to differences in infarct sizes [27]. Therefore, we investigated the effects of Et-1 on blood flow in normotensive and hypertensive rats. Since anesthetics are neuroprotective [10-12], a higher dose of Et-1 was required. We showed that 400 pmol was the optimal dose, because it produced the same infarct volume as the 200 pmol without anesthesia (data not shown). In the WKYRs, this dose led to an 80% reduction of the blood flow and after 60 minutes normal baseline values were reached. In the SHRs, Et-1 decreased the blood flow only by 65% and the normal values where reached after 50 minutes. Taken together these results indicate that the increased infarct volume in SHRs observed at 24 h is not due to a stronger decrease on blood flow by injection of Et-1.

Since glial cells are strongly involved in both induction of ischemic damage and neuroprotection [29], we also compared the activation of microglia and astrocytes in SHRs with their activation in WKY controls. Microglia are the first cells to respond to ischemic damage [30]. Although anti-CD68 stains two types of phagocytotic cells, activated microglia and infiltrated macrophages, it is unlikely that differences in CD68 staining are due to differences in macrophage infiltration. It has been well established in stroke models that until 72 h after the insult, macrophages represent only a small minority of CD68 positive cells [31]. For example, Shilling *et al.*[32] showed, using chimeric mice expressing green fluorescent protein-expressing leukocytes, that less than 10% of the CD68 positive cells during the first three days after the insult are hematogeneous macrophages. Since the infarct size was significantly larger in SHRs, we conclude that the reduced activation of microglial cells in these rats is not due to a lower level of ischemic damage. We hypothesize that microglial cells in SHRs are less susceptible to triggers produced during the ischemic cascade, such as ligands for the TLR-4 including heat-shock proteins, RNA and extra-cellular matrix proteins. To assess whether microglial cells in SHRs were less susceptible to activation via TLR-4, we injected different amounts of LPS into the striatum and measured the expression of CD68. LPS is a bacterial endotoxin inducing a strong inflammatory response via binding to the TLR-4 and interactions with CD14 and the lipopolysaccharide binding protein. TLR-4 and CD14 are expressed by microglia and ligation of these receptors leads to the activation and differentiation of microglia towards phagocytotic cells and subsequent production of neurotrophic factors, pro- inflammatory cytokines via NF-кB [23,24,33].

Since administration of small amounts of LPS induced a weaker expression of CD68 positive cells in SHRs compared to WKYRs, we postulated that microglia in SHRs are less susceptible for stimulation via TLR-4 at suboptimal levels of ligands. Infiltration and activation of monocytes within 24 h has been established in the LPS model for neuroinflammation [34] and, therefore, macrophages could be partially responsible for differences in CD68 expression between the two stains. In order to allow morphological identification of activated microglia, we also stained our slices from rats injected with 2 μg LPS with anti-Iba-1. Since the density of activated microglia showing increased Iba-1 binding and thickened processes was significantly increased in SHRs compared to WKYRs, we conclude that microglia in SHR rats are less sensitive to activation. The reduced iNOS expression in glial cells from (not stained with Cresyl Violet) LPS-treated SHRs is in accordance with this idea and suggests that this phenomenon leads to a diminution of neuroinflammation or oxidative stress. It remains to be established whether the increased expression of iNOS in neurons contributes to the increased susceptibility of SHRs to ischemic stroke at 24 h.

Based on the reduced levels of CD68 positive cells and both Iba-1 positive subsets, we also conclude that the total number of activated microglia in SHRs after injection of LPS is reduced compared to WKYRs.

We conclude that reduced sensitivity for microglial activation may indeed explain the reduced expression of CD68 in SHRs using the Et-1 model for stroke. Further experiments are required to determine whether differences in TLR expression or signaling are responsible for the reduced susceptibility of microglia in hypertensive rats. Our observation that cerebral ischemia evoked a lower level of CD68 expression in SHRs compared to WKYRs is in contrast to the findings of Marks *et al.*[26], who found that SHRSPs showed a more pronounced microglial response to focal cerebral ischemia compared to WKYRs in a permanent MCA occlusion model. In that model, blood flow reduction is more severe compared to our Et-1 model and permanent occlusion leads to a stronger ischemic insult and possibly stronger signals for microglial activation. This could explain, in part, the discrepancy between the results of Marks *et al.*[26] and our observation because we showed that only low levels of LPS exert different effects in SHRs and WKYRs. It is intriguing to speculate that the decreased microglial activation is responsible for the increase in infarct sizes at 24 h after stroke. Indeed, in addition to negative effects (for example, via induction of neuroinflammation), microglia also exert beneficial effects including phagocytosis of death cells, reuptake of excitotoxic neurotransmitters and production of neurotrophic factors and anti-inflammatory cytokines (for example, IL-10 and TGF-β) [19-22]. Lalancette-Hebert *et al.*[35] even found that when microglia were completely ablated, the infarct volume increased.

Activated microglia release a variety of substances, such as cytokines, which can lead to activation of astrocytes. Ischemia activates astrocytes resulting in an increasing expression of GFAP, which is also called reactive gliosis [20,21,36,37]. In contrast to CD68, GFAP expression in SHRs was not different compared to normotensive controls. However, we cannot exclude the possibility that astrocytes of SHRs are more responsive to stimuli (see Figure 3B), but that the levels of stimulatory factors, for instance, released by microglia is reduced. Data describing the differences in activation of astrocytes in normotensive and hypertensive rats after stroke are scarce, but it has been shown that there is less GFAP expression in the putamen and cortex of young SHRs until six months of age. Less astrocytes may be present in these brain areas, but it is also possible that a similar number of cells are present, but that the morphology is changed [38]. At six months of age, a significant increase of GFAP mRNA and an increase of GFAP immunoreactivity were demonstrated in different brain areas of SHRs compared to WKYRs [39].

We conclude that the Et-1 SHR model is a good preclinical model for stroke, meeting the STAIR criteria. This model allows testing of new therapies in conscious animals with hypertension as a co-morbidity factor. The difference in infarct size between normotensive and hypertensive rats we observed using Et-1 to induce the insult is in line with observations in other studies using other methods to induce ischemia [9,25,26]. Another advantage of this model is that it includes spontaneous reperfusion comparable to the clinical situation and that the technique is less invasive compared to the other models. For instance, in contrast to the filament model [27,28], the Et-1 model does not lead to mechanical damage of the endothelial layer of the vessel walls. Despite the fact that the exact location of the guide strongly influences the extent of blood flow reduction, we show that the infarct sizes are reproducible [27]. We also found that in the sham operated group, the activation of microglia and astrocytes remained low, at least until 72 h after the insult, indicating that the implantation of the guide alone hardly leads to inflammation within this time frame. A disadvantage of the Et-1 model is that the reduction in blood flow induced by a certain amount of Et-1 may be strain dependent. These differences in sensitivity to Et-1 should be taken into account when studying the impact of co-morbidity factors, like hypertension or diabetes, on treatments by comparing the efficacy of these treatments in different rat strains.

## Conclusions

Using the Et-1 model for ischemic stroke, we found that microglia of hypertensive rats show a reduced susceptibility to activation by LPS. Reduced microglial activation in SHRs was also observed after induction of cerebral ischemia by Et-1. This difference is not due to reduced infarct sizes, because infarcts were shown to be slightly increased (24 h) or equal in SHRs. The reduced susceptibility to microglial activation may be relevant with respect to preclinical testing of stroke therapies, because many potential drugs interact with microglia.

## Abbreviations

APES, 3-aminopropyltriethoxylane; Et-1, Endothelin-1; GFAP, Glial fibrillary acidic protein; iNOS, Inducible nitric oxide synthase; Iba-1, Ionized calcium binding adaptor molecule 1; LPS, Lipopolysaccharide; MCA, Middle cerebral artery; Min, Minutes; NDS, Neurological deficit score; PBS, Phosphate buffered saline; SHR(s), Spontaneously hypertensive rat(s); SPSHR(s), Stroke prone spontaneously hypertensive rat(s); STAIR, Stroke Therapy Academic Industry Round table; TLR, Toll like receptor; WKYR(s), Wistar Kyoto rat(s).

## Competing interests

The authors declare no competing interests.

## Authors’ contributions

DDG, SS, TZ, JDK and RK contributed to the design of the study. DDG and WS were involved in data acquisition. Statistical analysis was done by DDG, assisted by YM and RK. The draft of the manuscript was written by DDG and all authors contributed to the editing of the manuscript. All authors read and approved the final manuscript.
